# Melanin concentration and depolarization metrics measurement by polarization-sensitive optical coherence tomography

**DOI:** 10.1038/s41598-020-76397-4

**Published:** 2020-11-11

**Authors:** Masahiro Yamanari, Mutsuki Mase, Ryo Obata, Mitsuhiro Matsuzaki, Takahiro Minami, Seiji Takagi, Motoshi Yamamoto, Noriko Miyamoto, Koji Ueda, Naoshi Koide, Tadao Maeda, Kota Totani, Nobuyori Aoki, Yasuhiko Hirami, Satoshi Sugiyama, Michiko Mandai, Makoto Aihara, Masayo Takahashi, Satoshi Kato, Yasuo Kurimoto

**Affiliations:** 1 Engineering Department, Tomey Corporation, Nagoya, Aichi Japan; 2 Department of Ophthalmology, Graduate School of Medicine and Faculty of Medicine, The University of Tokyo, Tokyo, Japan; 3 Department of Ophthalmology, Kobe City Eye Hospital, Kobe, Hyogo Japan; 4 Department of Ophthalmology, Kobe City Medical Centre General Hospital, Kobe, Hyogo Japan; 5 Laboratory for Retinal Regeneration, Riken Centre for Biosystems Dynamics Research, Kobe, Hyogo Japan; 6 Vision Care Inc., Kobe, Hyogo Japan

**Keywords:** Imaging and sensing, Diagnostic markers

## Abstract

Imaging of melanin in the eye is important as the melanin is structurally associated with some ocular diseases, such as age-related macular degeneration. Although optical coherence tomography (OCT) cannot distinguish tissues containing the melanin from other tissues intrinsically, polarization-sensitive OCT (PS-OCT) can detect the melanin through spatial depolarization of the backscattered light from the melanin granules. Entropy is one of the depolarization metrics that can be used to detect malanin granules in PS-OCT and valuable quantitative information on ocular tissue abnormalities can be retrived by correlating entropy with the melanin concentration. In this study, we investigate a relationship between the melanin concentration and some depolarization metrics including the entropy, and show that the entropy is linearly proportional to the melanin concentration in double logarithmic scale when noise bias is corrected for the entropy. In addition, we also confirm that the entropy does not depend on the incident state of polarization using the experimental data, which is one of important attributes that depolarization metrics should have. The dependence on the incident state of polarization is also analyzed for other depolarization metrics.

## Introduction

Polarization-sensitive optical coherence tomograpy (PS-OCT) is one of the functional extensions of OCT to measure and analyze the polarimetric properties of the biological tissues^1^. Recently, technologies and applications of PS-OCT have grown as indicated by the rising trend of publications^2^. One of the promising applications of PS-OCT is detection of melanin in biological tissues. Particularly, it has been shown that the melanin in the retinal pigment epithelium (RPE) randomizes the state of polarization^3–5^. To visualize the randomness, degree of polarization uniformity (DOPU) has been developed and successfully utilized for retinal imaging^6,7^. Baumann et al. showed that DOPU monotonically increased as decreasing the concentration of melanin suspension^8^.

Although DOPU is a quantitative metric, it is a statistical measure of output state of polarization in response to a certain incident state of polarization and therefore covers only a portion of the polarimetric property. Lippok et al. showed that DOPU of a rubber phantom and melanin depended on the incident state of polarization^9,10^, and it was suggested that precaution was required to interpret DOPU and that other depolarization metrics were necessary to overcome the issue. Makita et al. demonstrated averaging of DOPU for two orthogonal incident states of polarization^11^, however its effect to the dependence on the incident state of polarization is unknown.

To make the depolarization metrics insensitive to the incident state of polarization, the depolarization metrics should be defined from Mueller matrix or its equivalent matrix, e.g., 4 $\times$ 4 covariance matrix, because these matrices represent the complete polarimetric properties including depolarization of a target, irrespective to the incident state of polarization. Lippok et al. demonstrated that depolarization index^9^, which was derived from Mueller matrix^12^, was insensitive to the incident state of polarization in PS-OCT. Other metrics such as depolarization power^13^, delta max^10,14^, and Lorentz depolarization indices^15^, decomposed from Mueller matrix are also known^16^. Entropy is another depolarization metric derived from the 4 $\times$ 4 covariance matrix^17^, and was demonstrated in PS-OCT for the anterior eye segment and the retina^18,19^. However, the dependence of these metrics derived from Mueller or 4 $\times$ 4 covariance matrices on the melanin concentration has not been investigated yet.

The purpose of this study is twofold. First, we investigate a relationship between the melanin concentration and the depolarization metrics, including the entropy. Second, using the same experimental data, we confirm that the depolarization metrics follow the attributes expected from theory with respect to the incident state of polarization.

## Results

### Melanin suspension

**Figure 1:**
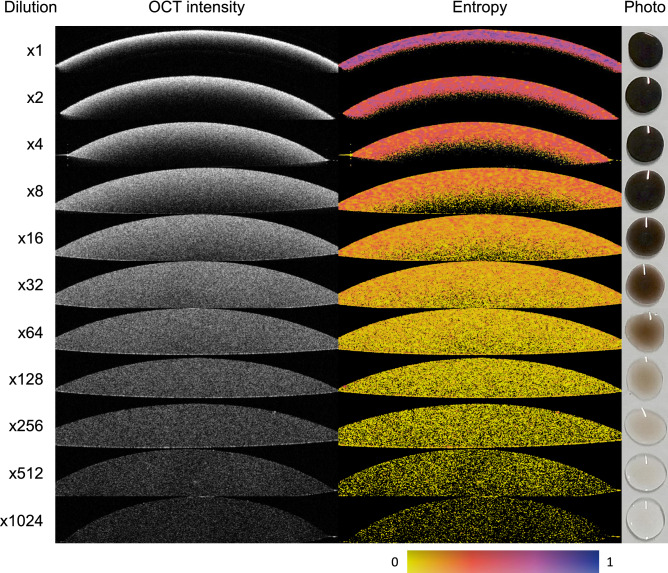
OCT intensity, entropy and photo of the melanin suspension at each dilution are shown. The entropy images in this figure were calculated from local Jones matrix with noise-bias correction, namely, $H_{{\text{L}}\_{\text{NBC}}}$ (see “Methods”). B-scan images were cropped from the original B-scans for visualization. An image size of the OCT B-scans was 0.76 $\times$ 6.00 mm (axial $\times$ lateral) in tissue. No averaging of B-scans was applied.

We made highly dense melanin suspension by applying vacuum filtration to natural eumelanin. Various concentrations were prepared with diluting the suspension. Droplets of the suspension on a glass slide were measured by a prototype of PS-OCT at 1050 nm wavelength. See “Methods” for details of the preparation of the melanin suspension, PS-OCT system and signal processing. Figure 1 showed OCT intensity, entropy images and photos of the droplets at various dilution ratios from $\times 1$ to $\times 1024$. The OCT intensity was highest at $\times 1$ dilution and decreased with increasing the dilution ratio. The penetration of the light in the OCT intensity images was lowest at $\times 1$ dilution ratio and increased with increasing the dilution ratio. The entropy was highest at $\times 1$ dilution and decreased with increasing the dilution ratio. These relations were confirmed in the diagram of droplets qualitatively, where the transparency of the droplet increased by increasing the dilution ratio.

**Figure 2:**
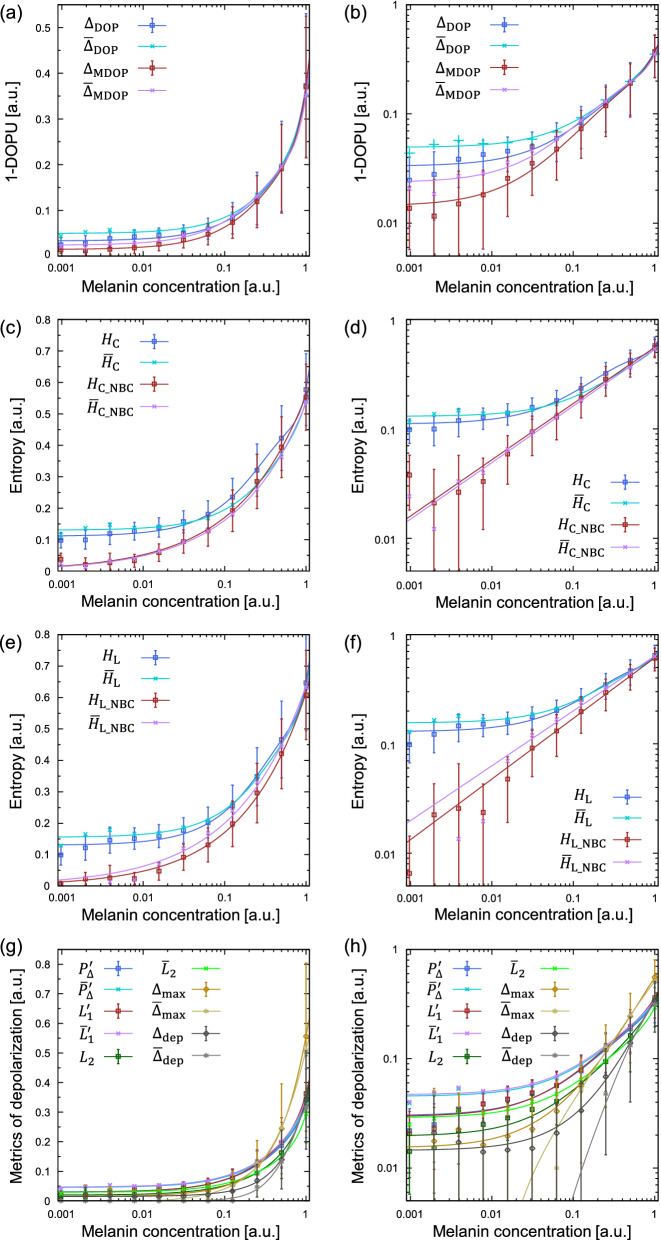
Plots of melanin concentration versus various depolarization metrics. The degree of polarization (**a**,**b**), the entropy calculated from the cumulative Jones matrix (**c**,**d**), the entropy calculated from the local Jones matrix (**e**,**f**), and some selected metrics (**g**,**h**) are shown. In all plots, the melanin concentration is shown with logarithmic scale. The plots of the left and right columns have linear and logarithmic vertical scales, respectively. Third-order polynomial fitting was applied to all the plots except for $H_{{\text{C}}\_{\text{NBC}}}$, ${\overline{H}}_{{\text{C}}\_{\text{NBC}}}$, $H_{{\text{L}}\_{\text{NBC}}}$ and ${\overline{H}}_{{\text{L}}\_{\text{NBC}}}$.

**Figure 3:**
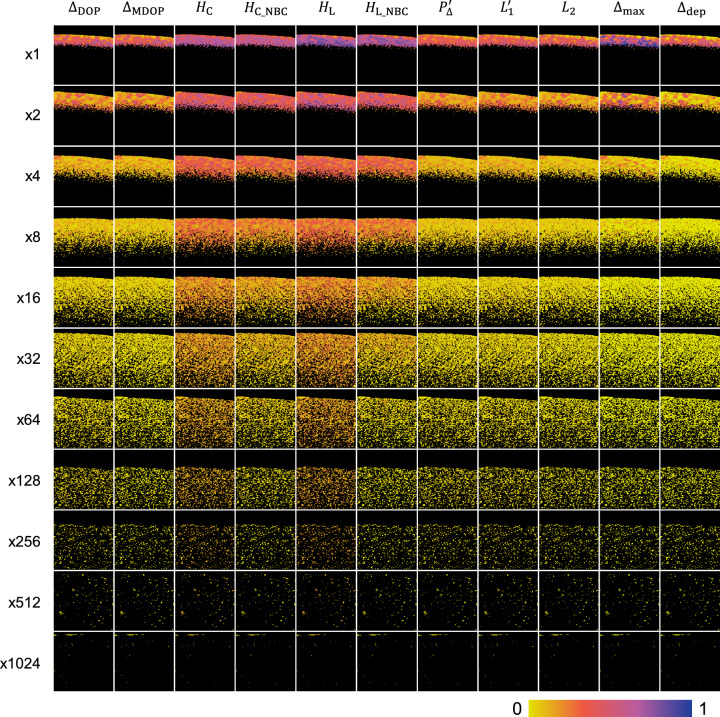
Cropped images of the depolarization metrics (columns) at the diluted ratios from $\times$ 1 to $\times$ 1024 (rows). The images were masked by black color if the signal-to-noise ratio was less than 15 dB. The cropped image size was 0.805 $\times$ 1.055 mm (axial $\times$ lateral) in tissue. No averaging of B-scans was applied.

**Figure 4:**
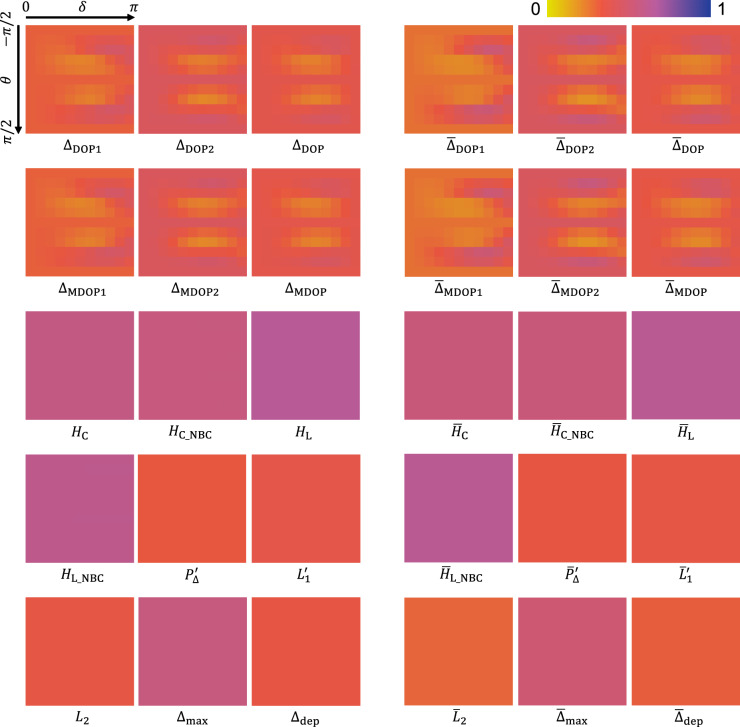
Visualization of the depolarization metrics in response to the virtual variable waveplate that was placed at the incident path of the illumination in the numerical simulation. Common raw data of the melanin suspension at $\times$1 diluted ratio were used. For each depolarization metric, both the phase retardation $\delta$ and the rotation angle $\theta$ were changed from 0 to $\pi$ rad and from $-\pi /2$ to $\pi /2$ with $\pi /10$ rad steps and shown as horizontal and vertical axes, respectively. As a result, the results of 11 $\times$ 11 conditions were visualized for each depolarization metric.

**Figure 5:**
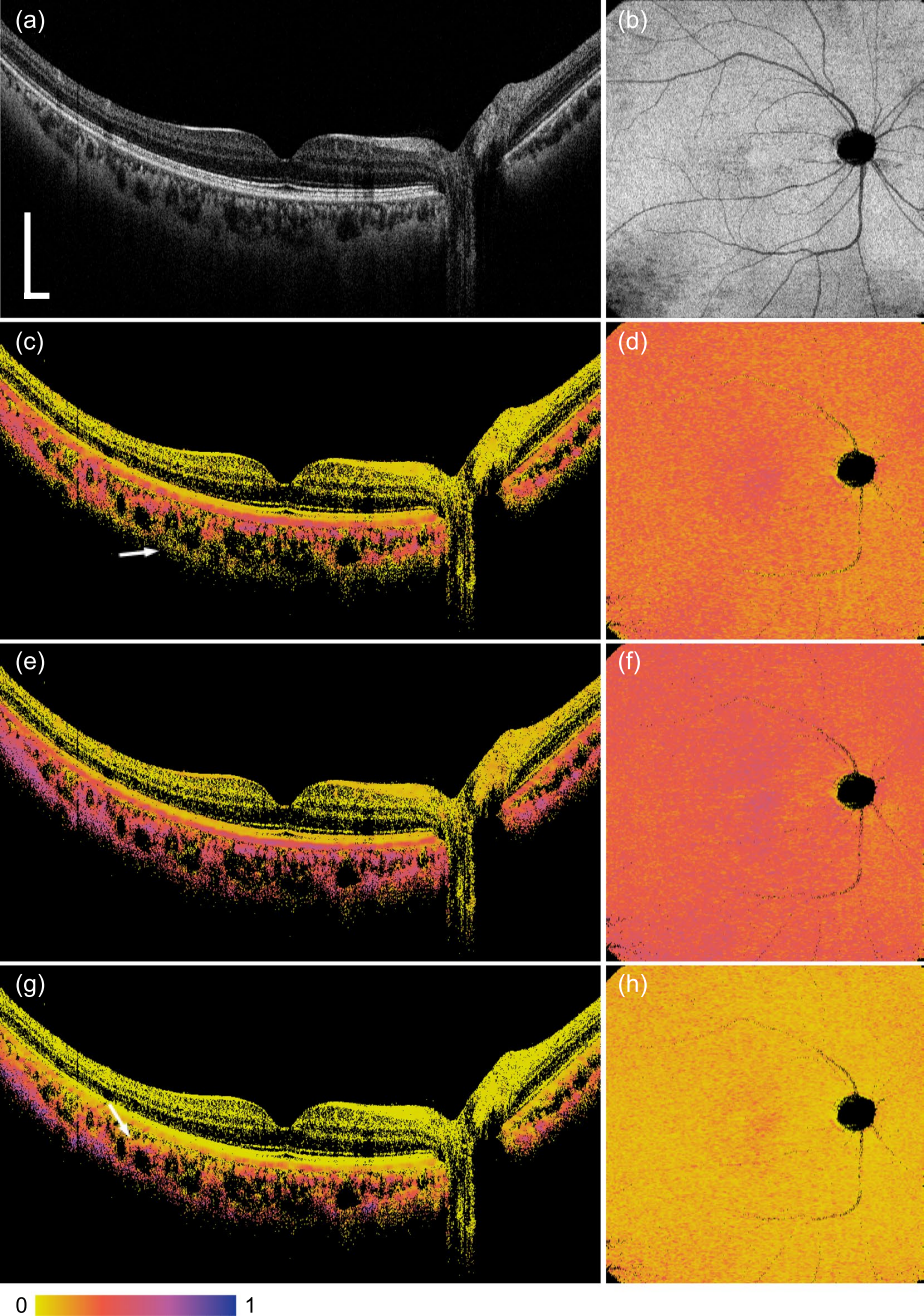
Images of the human retina in vivo. A B-scan of OCT intensity (**a**), *en face* OCT intensity at the RPE (**b**), a B-scan of entropy $H_{{\text{L}}\_{\text{NBC}}}$ (**c**), *en face* entropy $H_{{\text{L}}\_{\text{NBC}}}$ at the RPE (**d**), a B-scan of entropy $H_{{\text{C}}\_{\text{NBC}}}$ (**e**), *en face* entropy $H_{{\text{C}}\_{\text{NBC}}}$ at the RPE (**f**), a B-scan of DOPU $\Delta _{\text{MDOP}}$ (**g**), *en face* DOPU $\Delta _{\text{MDOP}}$ at the RPE (**h**) are shown. Scale bars of the B-scans and the *en face* images indicate 500 $\upmu$m and 1 mm, respectively. Because the B-scan images (**c**,**e**,**g**) were thresholded by the intensity to mask the noise floor by a black color and segmented pixels of these images at the RPE, rather than raw data of the depolarization metrics, were shown in (**d**,**f**,**h**), shadowed regions at large blood vessels and optic nerve head were depicted with the black color in (**d**,**f**,**h**). No averaging of B-scans was applied.

**Figure 6:**
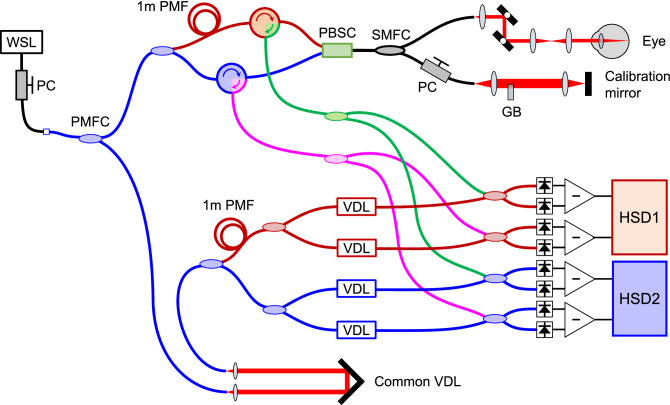
A schematic of the PS-OCT setup. The abbreviations are in the following; *WSL* wavelength-swept laser, *PC* polarization controller, *PMFC* polarization-maintaining filter coupler, *PMF* polarization-maintaining fiber, *PBSC* polarization beamsplitter/combiner, *GB* glass block, *VDL* variable delay line, *HSD* high-speed digitizer. Black and colored fibers indicate single-mode fiber (SMF) and PMF, respectively. For the measurement of the melanin suspension, the eye was replaced with a mirror that directed the light downward, a relay lens and the sample on the glass slide.

### Melanin concentration and depolarization metrics

For each B-scan data of the melanin suspension, Jones matrices in a region of $1.03 \times 3.60$ $\text{mm}^2$ that did not include a surface of the glass slide were converted to Stokes parameters or $4\times 4$ covariance matrices, and they were ensemble averaged. We then calculated various depolarization metrics. DOPU showed a spatial purity of Stokes parameters derived from measured Jones vectors. It was converted to randomness as 1-DOPU in this study, and denoted as $\Delta _{\text{DOP}}$. 1-DOPU with noise-bias correction^11^ was denoted as $\Delta _{\text{MDOP}}$. The entropy showed spatial randomness of 4 $\times$ 4 covariance matrices derived from measured Jones matrices^18^. When the entropy was calculated from the measured Jones matrix, it had inherently cumulative effect along the depth and was called cumulative Jones matrix in this paper. The entropy without and with the noise-bias correction in the cumulative Jones matrix was denoted as $H_{\text{C}}$ and $H_{\text{C}}\_{\text{NBC}}$, respectively. When the entropy was calculated from axially localized Jones matrix, it was derived by multiplying the measured Jones matrix by an inverse of the measured Jones matrix with an axial separation of 2 pixels and was called local Jones matrix in this paper. The entropy without and with the noise-bias correction in the local Jones matrix was denoted as $H_{\text{L}}$ and $H_{\text{L}}\_{\text{NBC}}$, respectively. Depolarization index was one of measures that represented polarimetric purity derived from Mueller matrices^12^, and was denoted as $P_\Delta$. It was converted as $P'_\Delta =1-P_\Delta$ to show randomness. First and second Lorentz depolarization indices, $L_1$ and $L_2$, were other definitions that represented polarimetric purity and randomness derived from Mueller matrices, respectively^15^. $L_1$ was converted to be randomness as $L'_1=1-L_1$ in this study. $\Delta _{\text{max}}$ and depolarization power $\Delta _{\text{dep}}$ were also other definitions that represented polarimetric randomness^13,14^. See “Methods” for the details of these metrics. These depolarization metrics were denoted without overlines. In addition, to evaluate ensemble averaging of the depolarization metrics, we calculated Stokes parameters or $4\times 4$ covariance matrices in a moving window with a kernel size of $5\times 11$ pixels ($22\times 64$ $\upmu \text{m}^2$ in axial $\times$ lateral directions), created B-scan images of the depolarization metrics and ensemble averaged the B-scan images of the depolarization metrics in the region of $1.03 \times 3.60$ $\text{mm}^2$ that did not include the surface of the glass slide. These ensemble averaged depolarization metrics were denoted with overlines.

Figure 2 showed various plots of the depolarization metrics in response to melanin concentration, which was inverse of the dilution ratio. In Fig. 2a,b, all metrics of DOPU monotonically decreased with decreasing the melanin concentration. In Fig. 2b, the slopes of all plots were close to linear at the melanin concentration greater than $\sim$0.06 and gradually decreased as the melanin concentration decreased. Noise-bias corrected $\Delta _{\text{MDOP}}$ and ${\overline{\Delta }}_{\text{MDOP}}$ had lower values at the melanin concentration less than $\sim$0.1 compared to $\Delta _{\text{DOP}}$ and ${\overline{\Delta }}_{\text{DOP}}$. $\Delta _{\text{DOP}}$ and ${\overline{\Delta }}_{\text{DOP}}$ had a plateau region at the melanin concentration less than $\sim$0.02.

Similar results were obtained in the case of entropy as shown in Fig. 2c–f. In both the entropies derived from cumulative non-local and local Jones matrices, noise-bias corrected entropies $H_{{\text{C}}\_{\text{NBC}}}$, ${\overline{H}}_{{\text{C}}\_{\text{NBC}}}$, $H_{{\text{L}}\_{\text{NBC}}}$, and ${\overline{H}}_{{\text{L}}\_{\text{NBC}}}$ had lower values at the melanin concentration less than $\sim$0.1 compared to $H_{\text{C}}$, ${\overline{H}}_{\text{C}}$, $H_{\text{L}}$, and ${\overline{H}}_{\text{L}}$. Notably, the relationship between the melanin concentration and the noise-bias corrected entropies $H_{{\text{C}}\_{\text{NBC}}}$, ${\overline{H}}_{{\text{C}}\_{\text{NBC}}}$, $H_{{\text{L}}\_{\text{NBC}}}$, and ${\overline{H}}_{{\text{L}}\_{\text{NBC}}}$ were close to linear in the double logarithmic plots in Fig. 2d,f. We therefore applied least squares fit to the linear data with $y = 10^b x^a$, where *x* and *y* denoted the melanin concentration and the entropy, respectively. It had a linear relation in a logarithmic scale as $\text{log}(y) = a \text{log}(x) + b$. We then obtained the results of *a* as 0.52, 0.52, 0.56, and 0.52 and *b* as -0.25, -0.27, -0.21, and -0.19 for $H_{{\text{C}}\_{\text{NBC}}}$, ${\overline{H}}_{{\text{C}}\_{\text{NBC}}}$, $H_{{\text{L}}\_{\text{NBC}}}$, and ${\overline{H}}_{{\text{L}}\_{\text{NBC}}}$, respectively. All of the noise-bias corrected entropies were therefore approximately in proportion to square roots of the melanin concentration.

We also plotted the results of the depolarization index, first and second Lorentz depolarization indices, $\Delta _{\text{max}}$ and depolarization power in Fig. 2g,h. These metrics also monotonically decreased with decreasing the melanin concentration. Similar to the DOPU and the entropy without the noise-bias correction, slopes of these metrics in the double logarithmic plot of Fig. 2h were close to linear at the melanin concentration greater than $\sim$0.1 and gradually decreased as the melanin concentration decreased. Note that $\Delta _{\text{dep}}$ and ${\overline{\Delta }}_{\text{dep}}$ at low melanin concentration were excluded from their plots in Fig. 2h, because they reached 0 at low melanin concentration, where their logarithm was undefined.

In addition, we also applied least squares fit to the linear data of all the depolarization metrics with $y = 10^b x^a$, and the results were plotted in Supplementary Fig. S1. The resultant parameters and the residual sum of squares (RSS) were shown in Supplementary Table S1. $\Delta _{\text{MDOP}}$ and ${\overline{\Delta }}_{\text{MDOP}}$ had low RSS, but they had upward discrepancy from the fitting curves at the low melanin concentration. $H_{{\text{C}}\_{\text{NBC}}}$, ${\overline{H}}_{{\text{C}}\_{\text{NBC}}}$, and $H_{{\text{L}}\_{\text{NBC}}}$ had the RSS less than 0.001, and they followed the fitting curves well. Although ${\overline{H}}_{{\text{L}}\_{\text{NBC}}}$ also followed the fitting curves, it had higher RSS because of high residuals at the low melanin concentration. This might be attributed to induced susceptibility to noise in the localizing operation to the Jones matrix. Although ${\overline{\Delta }}_{\text{max}}$ and ${\overline{\Delta }}_{\text{dep}}$ also had the RSS less than 0.001, they had plateau region at the low melanin concentration.

To illustrate the relationship between the melanin concentration and depolarization metrics, cropped images of the depolarization metrics were tiled in Fig. 3. It corresponded to the results in Fig. 2 well. At the concentration of 1, all entropies and $\Delta _{\text{max}}$ showed relatively higher values (>0.6) than the other metrics. Compared to $\Delta _{\text{max}}$, which decreased steeply as decreasing the concentration, the entropies decreased gradually. Furthermore, the noise-bias correction of $H_{{\text{C}}\_{\text{NBC}}}$ and $H_{{\text{L}}\_{\text{NBC}}}$ was effective to keep the decreasing trend of the entropy at dilution ratios over $\times 8$, whereas $H_{\text{C}}$ and $H_{\text{L}}$ were mostly in a plateau region at dilution ratios over $\times 8$. The effect of the noise-bias correction in $\Delta _{\text{MDOP}}$ compared to $\Delta _{\text{DOP}}$ was not prominent as in the case of the entropies. In other words, although $H_{\text{C}}$ and $H_{\text{L}}$ were more susceptible to the noise at the low concentration compared to $\Delta _{\text{DOP}}$ and probably also other depolarization metrics, the entropies showed better contrast that the entropies decreased monotonically at the low concentration once the noise-bias correction was applied as indicated by $H_{{\text{C}}\_{\text{NBC}}}$ and $H_{{\text{L}}\_{\text{NBC}}}$.

### Influence of the incident state of polarization

When all elements of Jones matrix were measured, it was possible to investigate the influence of the incident state of polarization to the depolarization metrics with numerical simulation. To reduce the degree of freedom in the simulation, it is reasonable to introduce an assumption based on the experimental results by Lippok et al. that DOPU depends on the ellipticity of the incident polarization^10^. We assume that the melanin granules are randomly oriented in the target and the randomness of the backscattered state of polarization from the melanin granules is independent from the orientation of the incident polarization but is dependent on the ellipticity of the incident polarization. Under this assumption, rotating a virtual variable linear retarder ${\mathbf {J}}_{\text{R}}(\theta ,\delta )$ in Eq. (23), where $\theta$ and $\delta$ denote the rotation angle and the phase retardation, respectively, is sufficient to investigate whether the ellipticity of the incident polarization influences the depolarization metrics or not.

Figure 4 showed the depolarization metrics with the virtual variable linear retarder ${\mathbf {J}}_{\text{R}}(\theta ,\delta )$. We changed $\theta$ and $\delta$ in ranges of $-\pi /2 \le \theta \le \pi /2$ and $0 \le \delta \le \pi$, respectively, with $\pi /10$ steps. The raw data were same as the $\times 1$ dilution ratio shown in Figs. 1-3. Although all the parameters of DOPU depended on ${\mathbf {J}}_{\text{R}}(\theta ,\delta )$, the other parameters did not depend on it. Minimum and maximum standard deviations of the DOPU parameters were 0.048 and 0.081, respectively. In contrast, standard deviations of the other metrics were less than 0.001. To understand the worst-case scenario, we calculated a difference between the maximum and minimum, defined as $\Lambda$, for each depolarization metric. $\Lambda$ of the DOPU parameters ranged from 0.226 to 0.362. In contrast, $\Lambda$ of the other parameters was less than 0.007. Figure 4 and the numerical results described above clearly demonstrated the advantage of the depolarization metrics defined from $4\times 4$ covariance matrix or Mueller matrix over the DOPU parameters.

## Discussion

In Fig. 2 overall, there was no major difference between two methods of the ensemble averaging, namely, averaging of the Stokes parameters or $4\times 4$ covariance matrices and averaging of the depolarization metrics, except for small drifts. It is therefore possible to use both calculation methods for quantitative evaluation of the melanin concentration unless same calculation method is used throughout the quantitative comparison.

In Fig. 4, in addition to $\Delta _{\text{DOP1}}$, $\Delta _{\text{DOP2}}$, $\Delta _{\text{MDOP1}}$ and $\Delta _{\text{MDOP2}}$, which were previously shown depending on the incident state of polarization^10^, $\Delta _{\text{DOP}}$ and $\Delta _{\text{MDOP}}$ also showed the dependence on the incident state of polarization as well. Therefore, the averages of DOPU for two orthogonal incident states of polarization were not effective to cancel the dependence on the incident state of polarization. This is because a randomness of a relative phase between the first and second columns of measured Jones matrix is not involved in DOPU. If the incident state of polarization is uncontrolled, measured DOPU would have the highest variability. This can be applied to PS-OCT which has SMF in its sample arm. If the incident state of polarization is controlled to be a circular polarization, the variability would be greatly reduced. In the case of retinal imaging, however, the incident state of polarization can be changed by the birefringence of ocular media, such as a cornea^20^ and a retinal nerve fiber layer^21^. This would induce a certain variability of DOPU even if the probing light was set to be the circular polarization. To avoid the dependence on the incident state of polarization, the only way is to use the depolarization metrics that are derived from $4\times 4$ covariance matrix or Mueller matrix and that are modeled to be independent from the incident state of polarization.

Note that if the entropy is derived from 2 $\times$ 2 covariance matrix, it also depends on the incident state of polarization as demonstrated by Lippok et al.^14^ When the entropy is derived from 4 $\times$ 4 covariance matrix as in our case^18^, it does not depend on the incident state of polarization. In general, the definition of entropy can vary depending on one’s purpose^22^. The natural scalability of the entropy defined in a von Neumann sense is one of attributes that the other depolarization metrics do not have. In the field of radar polarimetry, the entropy is often derived from $3\times 3$ coherency matrix, whose degree of freedom is decreased because of the reflection symmetry, and has been used for classifying land objects^23–26^.

The entropy used in our study has several technical advantages. First, it is insensitive to the incident state of polarization as we have discussed. Second, the noise-bias correction is available, which is important particularly for the detection of low melanin concentration as shown in Fig. 2. Third, a mitigation of the underestimation of the entropy, called asymptotic quasi maximum likelihood estimator^27^, is implemented. It is effective when a number of samples for ensemble averaging of $4\times 4$ covariance matrices is low^18^. Only the entropy has all of these features among the depolarization metrics. In addition, Figs. 2 and 3 showed that the noise-bias-corrected entropy could measure the melanin suspension in a wide range of the concentration without plateau regions. These features of the entropy encourages us to use the entropy for the evaluation of the melanin in biological tissues. On the other hand, a disadvantage of the entropy would be that the additional complexity is required for the interferometer to measure all elements of Jones matrix. The depolarization metrics that can be derived from statistical assessment of measured Jones vector, such as DOPU and entropy derived from $2\times 2$ covariance matrix, have an advantage that the interferometer to measure Jones vector can be made simple relatively. These metrics would be reasonably useful when the dependence on the incident state of polarization does not critically impact on the imaging and quantification as proved by a lot of clinical studies^2,7^. Although the contrast of the entropy at low melanin concentration would be better than that of the DOPU as shown in Fig. 2, the melanin concentration at the RPE and the choroid of healthy eyes is distributed in higher region that both the entropy and the DOPU can make the contrast, which can be seen in Fig. 5 that is described later. However, the better contrast of the entropy may be useful for images of pathological eyes. Further studies would be required to understand whether the entropy and the other depolarization metrics are also useful for clinical applications. Nevertheless, to be fair, our experimental condition was originally optimized by monitoring the entropy, and our results did not necessarily mean that the entropy was always the best for any experimental conditions and any samples.

Figure 5 shows retinal images of a human eye in vivo. The subject was a left eye of 38-year-old Japanese male with non-pathologic high myopia (− 6.5 D). Images of the same eye was demonstrated previously^19^. In Fig. 5c, the entropy $H_{{\text{L}}\_{\text{NBC}}}$ at the RPE and the choroid was high because of the melanin. The entropy $H_{{\text{L}}\_{\text{NBC}}}$ of the RPE at the fovea was slightly higher than the peripheral region of the fovea. It was also visible in the *en face* entropy $H_{{\text{L}}\_{\text{NBC}}}$ image at the RPE, which was qualitatively consistent with literature^28^ and previous observation using DOPU^29^. These properties were consistent in the entropy $H_{{\text{C}}\_{\text{NBC}}}$ images (e), (f) and the DOPU $\Delta _{\text{MDOP}}$ images (g), (h). Although the reason is not clear, however, there were minor but discernible differences between $H_{{\text{L}}\_{\text{NBC}}}$, $H_{{\text{C}}\_{\text{NBC}}}$, and $\Delta _{\text{MDOP}}$ in the choroid. Posterior or anterior regions of the choroid indicated by arrows in Fig. 5c or g partly showed lower value than other region of the choroid, respectively. In contrast, Fig. 5e did not show such regional difference in depth apparently. Further studies would be required to understand these properties in the images of the depolarization metrics. In addition, it is our future work to investigate more subjects of healthy and pathologic eyes using our PS-OCT. We recently demonstrated that the entropy could visualize the induced-pluripotent-stem-cell-derived RPE both in vitro and in vivo^30^, suggesting that PS-OCT would be promising for monitoring regenerated pigment cells in both laboratories and clinics for regenerative medicine.

A physical mechanism that the scattering from the melanin granule results in the spatial depolarization is still poorly understood. In the case of Mie scattering from spherical particles, it is known that the spectral response of the scattered light is modulated^31,32^ and that the depolarization is also observed when a density of the Mie scatterers is high^8^. Melanin granules, such as the melanosome and the melanolipofuscin, are nonspherical particles whose size is from 1 to a few $\upmu$m^8,33,34^, which is in the order of the wavelength as well as the Mie scatterers. In general, it is known that such nonspherical particles have unsteady scattering matrix even in simplified models^35^. It would further complicate the phenomenon that the scattering from the nonspherical particles are coherently summed in a spatial volume determined by axial and transversal point spread functions. These factors might contribute the depolarization of the scattered light from the melanin granules and might be understood only through a statistical way.

In the RPE, a number of melanosomes decreases with age and numbers of melanolipofuscin and melanolysosomes increase^33,36,37^. Melanosomes often have long and narrow spindle shapes and are thought to change into the melanolipofuscin and melanolysosomes with aging. Melanolipofuscins often have a shape that the elliptic melanin granule is enclosed by lipofuscin. Melanolysosomes are a fusion of the melanin granule and a lysosome and may only be identified by electron microscopy^38^. It does not seem that PS-OCT can distinguish these granules, which is therefore one of limitations of the PS-OCT imaging. It would also be true for the other imaging modalities that can detect the retinal melanin^39^. Nevertheless, it is of great interest how the depolarization metrics respond to the shape, size distribution, and absolute concentration of the melanin granules. These parameters of the melanin granules were measured by Baumann et al.^8^ and Harper et al.^32^ previously. Although the melanin granules were filtered by vacuum filtration to ensure the size of the melanin granules was less than 5 $\upmu$m in our study as described in “Methods”, it was one of our limitations that we did not measure these parameters of the melanin granules because of limited access to electron microscopy, particle size analyzer, and freeze-drying machine. In addition, in the case of PS-OCT, we need to distinguish the melanin granules from other depolarizing materials, such as hard exudates^40,41^. However, it would not be difficult as hard exudates are also visible in fundus photo.

In summary, we investigated the relationship between the melanin concentration and the depolarization metrics including the entropy. All the depolarization metrics monotonically decreased as decreasing the melanin concentration. Particularly, the entropies with noise-bias correction were approximately proportional to square roots of the melanin concentration without plateau region. Numerical analysis reproduced and showed that DOPU was sensitive to the incident state of polarization and that the other depolarization metrics were not. In addition, to the best of our knowledge, this is the first study comparing the responses of the various depolarization metrics measured by PS-OCT to the melanin concentration. Because the entropy with noise-bias correction maintained a contrast in a wide range of the melanin concentration, the entropy would be a promising depolarization metric. Along with our future clinical studies, the characteristic of the entropy may contribute to enhancing the clinical efficacy of PS-OCT.

## Methods

### Preparation of melanin suspension

One-gram natural eumelanin extracted from squid ink (Toyo ADL Corporation, Tokyo, Japan) was immersed into purified water of 100 ml. As the particle size of this eumelanin could not be controlled, we extracted the melanin granules that had a diameter less than 5 $\upmu$m with vacuum filtration. The filtered melanin suspension was precipitated using a centrifuge for 30 min. The precipitated suspension without the supernatant was extracted, diluted by purified water to be 3 ml, and mixed by a vortex mixer to avoid aggregation of the granules. It was centrifuged again, and the precipitated suspension was slightly diluted by purified water to be 60 $\upmu$l and mixed by a vortex mixer. This suspension was defined as dilution ratio of $\times 1$ in our study. Small portion of the suspension was dropped on a glass slide and used for the PS-OCT measurement. A portion of the remaining suspension that was not used for the previous measurement was diluted to be twice ($\times 2$) the volume by purified water. The process of the dilution was repeated for 10 times until the diluted volume reached to $\times 1024$ and the PS-OCT measurements were performed successively.

### PS-OCT system

We built a prototype of PS-OCT that measured all elements of Jones matrix as shown in Fig. 6. The light source was a tunable vertical cavity surface emmiting laser (VCSEL, Thorlabs Inc., Newton, New Jersey, US), which was demonstrated for high-speed and long-range swept-source OCT (SS-OCT) imaging of the retina^42,43^, with a center wavelength of 1050 nm, a bandwidth of 100 nm, and a repetition rate of 100 kHz. The interferometer was configured as parallel-detection PS-OCT (PD-PS-OCT)^19,44^ to measure all elements of Jones matrix without compromising the A-scan rate nor the axial depth range. In brief, a polarization-maintaining filter coupler (PMFC), which used miniaturized collimators and Wollaston prisms inside a package of the coupler, split the light to the reference and sample arms. In the sample arm, another PMFC further split the light and to distinguish two lights that were going to be two orthogonal incident states of polarization, 1 m polarization-maintaining fiber (PMF) was used for one of the output ports from the PMFC. These lights were combined by a polarization beamsplitter/combiner (PBSC) and directed to a 1/99 single-mode fused coupler (SMFC). 1% of the light illuminated a calibration mirror, which was used to stabilize the signal phase numerically^44^. 99% of the light illuminated the eye, and the backscattered light was directed to the PBSC and circulators. The lights directed by the circulators were further split by PMFCs and interfered with the reference lights that were configured to distinguish which light was from which input port of the PBSC in the sample arm. Each signal was detected by a balanced photoreceiver which was followed by a high-speed digitizer (HSD). In this way, we measured the Jones matrix of the sample in a parallel manner. The axial resolution and the depth range were 7.3 $\upmu$m and 4.49 mm in tissue, respectively. The measured Jones matrix was moving averaged with a kernel size of 3 $\times$ 1 (axial $\times$ lateral) pixels to reduce the speckle noise and to mitigate the bias at the boundaries^45^. The filtered Jones matrix was described as

1 $$\begin{aligned} {\mathbf {J}} = \left( \begin{array}{ccc} j_{11} &{} j_{12} \\ j_{21} &{} j_{22} \end{array} \right) . \end{aligned}$$

This filtered Jones matrix was used for all processings of the depolarization metrics.

For the measurement of the human eye, the experiment was conducted following the Declaration of Helsinki, approved by Tomey Corporation, and performed at Tomey Corporation. Informed consent was obtained from the volunteer.

### Depolarization metrics

Since DOPU is defined as a statistical measure of an output Jones vector for a certain input Jones vector^6^, two DOPUs can be calculated for the first and second columns of the Jones matrix^11^. We calculated two DOPUs and also averaged DOPU following Makita et al.^11^ as

2 $$\begin{aligned} \text{DOP1}= & {} \frac{\sqrt{{\overline{s}}^2_{11} + {\overline{s}}^2_{12} + {\overline{s}}^2_{13}}}{{\overline{s}}_{10}}, \end{aligned}$$

3 $$\begin{aligned} \text{DOP2}\,=\, & {} \frac{\sqrt{{\overline{s}}^2_{21} + {\overline{s}}^2_{22} + {\overline{s}}^2_{23}}}{{\overline{s}}_{20}}, \end{aligned}$$

4 $$\begin{aligned} \text{DOP}\,=\, & {} \tfrac{1}{2}( \text{DOP1} + \text{DOP2} ), \end{aligned}$$

where

5 $$\begin{aligned} \left[ \begin{array}{c} s_{n0}\\ s_{n1}\\ s_{n2}\\ s_{n3} \end{array} \right] = \left[ \begin{array}{c} |j_{1n}|^2 + |j_{2n}|^2\\  |j_{1n}|^2 - |j_{2n}|^2\\ 2\text{Re}[j_{1n}j_{2n}^*]\\  -2\text{Im}[j_{1n}j_{2n}^*] \end{array} \right] , \quad n=1,2, \end{aligned}$$

and the overline denoted spatial ensemble averaging. We defined $\Delta _{\text{DOP1}} \,=\, 1- \text{DOP1}$, $\Delta _{\text{DOP2}} = 1- \text{DOP2}$ and $\Delta _{\text{DOP}} = 1- \text{DOP}$ to make their 0 and 1 comparable to other depolarization metrics as nondepolarizing and depolarizing states, respectively. We also applied noise-bias correction for DOPU following Makita et al.^11^ as

6 $$\begin{aligned} \text{MDOP1}\,=\, & {} \frac{\sqrt{\overline{s'}^2_{11} + \overline{s'}^2_{12} + \overline{s'}^2_{13}}}{\overline{s'}_{10}}, \end{aligned}$$

7 $$\begin{aligned} \text{MDOP2}\,=\, & {} \frac{\sqrt{\overline{s'}^2_{21} + \overline{s'}^2_{22} + \overline{s'}^2_{23}}}{\overline{s'}_{20}}, \end{aligned}$$

8 $$\begin{aligned} \text{MDOP}\,=\, & {} \tfrac{1}{2}( \Delta _{\text{DOP1}} + \Delta _{\text{DOP2}} ), \end{aligned}$$

where

9 $$\begin{aligned} \left[ \begin{array}{c} s'_{n0}\\ s'_{n1}\\ s'_{n2}\\ s'_{n3} \end{array} \right] = \left[ \begin{array}{c} |j_{1n}|^2 + |j_{2n}|^2 - (|\xi _{1n}|^2 + |\xi _{2n}|^2)\\ |j_{1n}|^2 - |j_{2n}|^2 - (|\xi _{1n}|^2 - |\xi _{2n}|^2)\\ 2\text{Re}[j_{1n}j_{2n}^*]\\ -2\text{Im}[j_{1n}j_{2n}^*] \end{array} \right] , \qquad n=1,2, \end{aligned}$$

and $|\xi _{1n}|^2$ and $|\xi _{2n}|^2$ are the noise power of the first and second rows and *n*-th column element of the Jones matrix, respectively. Note that weighted sum of DOPUs in Makita et al.^11^ is simplified in Eqs. (4) and (8) because the SNRs of the first and second columns of the Jones matrix are almost same in PD-PS-OCT. We defined $\Delta _{\text{MDOP1}} = 1- \text{MDOP1}$, $\Delta _{\text{MDOP2}} = 1- \text{MDOP2}$ and $\Delta _{\text{MDOP}} = 1- \text{MDOP}$ to make their 0 and 1 comparable to other depolarization metrics as nondepolarizing and depolarizing states, respectively.

We calculated the entropy using Cloude-Pottier decomposition^18,25,26^ of the 4 $\times$ 4 covariance matrix derived from the Jones matrix. We also applied a bias correction of the eigenvalues for small number of sampling points^27^ and the other bias correction of the noise. Full details of the theory were described previously^18^. The entropies of the non-localized cumulative Jones matrix without or with the noise-bias correction, which were denoted as $H_{\text{C}}$ or $H_{{\text{C}}\_{\text{NBC}}}$ in this study, respectively, were described in Sect. 2.4 of Yamanari et al.^18^ Likewise, the entropies of the local Jones matrix without or with the noise-bias correction, which were denoted as $H_{\text{L}}$ or $H_{{\text{L}}\_{\text{NBC}}}$ in this study, respectively, were described in Sect. 2.5 of Yamanari et al.^18^.

Depolarization index was proposed by Gil and Bernabeu^12^. To calculate the depolarization index, we followed Sect. 5.11.3 of Gil and Ossikovski^16^. Equation (1) was vectorized as ${\underline{\kappa }} = \left[ j_{11}, j_{12}, j_{21}, j_{22} \right] ^{\text{T}}$, where the superscripted T denoted a transpose operation to the vector. The 4 $\times$ 4 covariance matrix ${\mathbf {T}}$ with ensemble averaging was derived as

10 $$\begin{aligned} {\mathbf {T}} = \overline{{\underline{\kappa }}{\underline{\kappa }}^\dagger }, \end{aligned}$$

where the superscripted dagger denotes complex transpose. The depolarization index $P_\Delta$ was defined as

11 $$\begin{aligned} P_\Delta = \frac{1}{\sqrt{3}} \sqrt{4 \sum _{n=0}^{3} {\hat{\lambda }}_n^2 -1} \qquad \left( {\hat{\lambda }}_n = \frac{\lambda _n}{\text{tr}{\mathbf {T}}} \right) , \end{aligned}$$

where $\lambda _n$ was *n*-th eigenvalue of ${\mathbf {T}}$ and tr denoted a trace of the matrix.

Lorentz depolarization indices were proposed by Ossikovski^15^. The 4 $\times$ 4 covariance matrix ${\mathbf {T}}$ was converted to Mueller matrix ${\mathbf {M}}$ whose element $m_{kl}$ was written as^16^

12 $$\begin{aligned} m_{kl} = \text{tr} \left( {\mathbf {E}}_{kl} {\mathbf {T}} \right) \qquad \left( k,l = 0,1,2,3 \right) , \end{aligned}$$

where ${\mathbf {E}}_{kl} = \mathbf {\sigma }_k \otimes \mathbf {\sigma }_l$, $\otimes$ denoted the Kronecker product and $\mathbf {\sigma }_{k,l}$ was the Pauli matrices plus the identity matrix denoted as

13 $$\begin{aligned} \mathbf {\sigma }_0 = \begin{pmatrix} 1 &{} 0 \\ 0 &{} 1 \end{pmatrix}, \quad \mathbf {\sigma }_1 = \begin{pmatrix} 1 &{} 0 \\ 0 &{} -1 \end{pmatrix}, \quad \mathbf {\sigma }_2 = \begin{pmatrix} 0 &{} 1 \\ 1 &{} 0 \end{pmatrix}, \quad \mathbf {\sigma }_3 = \begin{pmatrix} 0 &{} -i \\ i &{} 0 \end{pmatrix}. \end{aligned}$$

${\mathbf {N}}$-matrix was defined from ${\mathbf {M}}$ as

14 $$\begin{aligned} {\mathbf {N}} = {\mathbf {G}}{\mathbf {M}}^{\text{T}} {\mathbf {G}}{\mathbf {M}}, \end{aligned}$$

where the Minkowski metric matrix ${\mathbf {G}} = \text{diag}\left[ 1,-1,-1,-1\right]$ was used. The eigenvalues of ${\mathbf {N}}$, $\rho _n$ with $n=1,2,3,4$, were used to define the first and second Lorentz depolarization indices, $L_1$ and $L_2$ as

15 $$\begin{aligned} L_1\,=\, & {} \sqrt{\frac{\rho _2 + \rho _3 + \rho _4}{3\rho _1}}, \end{aligned}$$

16 $$\begin{aligned} L_2= & {} \sqrt{\frac{4\sum _{n=1}^{4} \rho _n^2 - \left( \sum _{n=1}^{4} \rho _n \right) ^2}{3 \left( \sum _{n=1}^{4} \rho _n \right) ^2}}. \end{aligned}$$

We defined

17 $$\begin{aligned} L'_1 = 1-L_1 \end{aligned}$$

to make $L'_1 = 0$ and 1 comparable to other depolarization metrics as nondepolarizing and depolarizing states, respectively.

Depolarization power $\Delta _{\text{dep}}$ was proposed by Lu and Chipman^13^. Similar to $\Delta _{\text{dep}}$, Lippok et al. developed $\Delta _{\text{max}}$, which was a depolarization metric along an axis of the maximum depolarization^10,14^. We followed them^13,14^ to calculate $\Delta _{\text{dep}}$ and $\Delta _{\text{max}}$. In brief, the diattenuation was removed from ${\mathbf {M}}$ and a new matrix $\mathbf {M'}$ was defined as

18 $$\begin{aligned} \mathbf {M'} = {\mathbf {M}}{\mathbf {M}}_D^{-1}, \end{aligned}$$

where ${\mathbf {M}}_D$ was a diattenuator Mueller matrix^13^. $\mathbf {M'}$ was further decomposed as

19 $$\begin{aligned} \mathbf {M'} = {\mathbf {M}}_\Delta {\mathbf {M}}_R = \begin{bmatrix} 1 &{} \vec {0}^{\text{T}} \\ \vec {P}_\Delta &{} {\mathbf {m}}_\Delta {\mathbf {m}}_R \end{bmatrix} \end{aligned}$$

where ${\mathbf {M}}_\Delta$ and ${\mathbf {M}}_R$ were depolarizing Mueller matrix and retarder Mueller matrix, respectively. $\vec {0}$ was the three-element zero vector. $\vec {P}_\Delta$ was the polarizance vector of ${\mathbf {M}}_\Delta$. ${\mathbf {m}}_\Delta$ and ${\mathbf {m}}_R$ were $3\times 3$ submatrices of ${\mathbf {M}}_\Delta$ and ${\mathbf {M}}_R$, respectively. The depolarization power $\Delta _{\text{dep}}$ was given by

20 $$\begin{aligned} \Delta _{\text{dep}} = 1 - \frac{\left| \text{tr}({\mathbf {m}}_\Delta ) \right| }{3} = 1 - \frac{\sum _{n=1}^{3} {\hat{\upsilon }}_n }{3}, \end{aligned}$$

where ${\hat{\upsilon }}_n = \upsilon / (\sum _{n=1}^3 \upsilon _n)$ and $\upsilon _n$ was eigenvalues of ${\mathbf {m}}_\Delta$. Because ${\mathbf {m}}_\Delta$ was a symmetric matrix, $\upsilon _n$ was non-negative. Similarly, $\Delta _{\text{max}}$ was given by

21 $$\begin{aligned} \Delta _{\text{max}} = 1 - \upsilon _{\text{min}}, \end{aligned}$$

where $\upsilon _{\text{min}}$ was a minimum of $\upsilon _n$ in $n=1,2,3$.

### Numerical simulation to change the incident state of polarization

The Jones matrix measured by our system can be described as

22 $$\begin{aligned} {\mathbf {J}}_{\text{measured}} = {\mathbf {J}}_{\text{out}} {\mathbf {J}}_{\text{sample}} {\mathbf {J}}_{\text{in}}, \end{aligned}$$

where ${\mathbf {J}}_{\text{in}}$ and ${\mathbf {J}}_{\text{out}}$ are Jones matrices of the incoming and outgoing paths including the optical fibers and ocular media, such as a cornea. ${\mathbf {J}}_{\text{sample}}$ is double-pass Jones matrix of the sample. Assuming that the diattenuation was negligible, all Jones matrices in Eq. (22) were unitary matrices and thus could be regarded as elliptic retarders. We multiply Jones matrix of a linear retarder ${\mathbf {J}}_{\text{R}}(\theta ,\delta )$ from right side of ${\mathbf {J}}_{\text{measured}}$ as

23 $$\begin{aligned} {\mathbf {J}}'_{\text{measured}} = {\mathbf {J}}_{\text{measured}} {\mathbf {J}}_{\text{R}}(\theta ,\delta ) = {\mathbf {J}}_{\text{out}} {\mathbf {J}}_{\text{sample}} {\mathbf {J}}_{\text{in}} {\mathbf {J}}_{\text{R}}(\theta ,\delta ), \end{aligned}$$

where

24 $$\begin{aligned} {\mathbf {J}}_{\text{R}}(\theta ,\delta )= & {} {\mathbf {R}}(\theta ) \begin{pmatrix} e^{-i\frac{\delta }{2}} &{} 0 \\ 0 &{} e^{+i\frac{\delta }{2}} \end{pmatrix} {\mathbf {R}}(-\theta ) \end{aligned}$$

25 $$\begin{aligned} {\mathbf {R}}(\theta )= & {} \begin{pmatrix} \cos \theta &{} -\sin \theta \\ \sin \theta &{} \cos \theta \end{pmatrix}. \end{aligned}$$

In Eq. (23), the incident state of polarization can be partially controlled by ${\mathbf {J}}_{\text{R}}(\theta ,\delta )$. If we change $\theta$ and $\delta$, the first and second columns of ${\mathbf {J}}_{\text{in}} {\mathbf {J}}_{\text{R}}(\theta ,\delta )$ can become Jones vectors of linear or circular polarizations at certain values of $\theta$ and $\delta$. We used ${\mathbf {J}}'_{\text{measured}}$ instead of ${\mathbf {J}}_{\text{measured}}$ for the further processing described in the above to obtain the depolarization metrics, and obtained the results shown in Fig. 4.

## Supplementary information

Supplementary material 1
