# Supplementary material for: Melanin concentration and depolarization metrics measurement by polarization-sensitive optical coherence tomography
Source: Sci Rep. 2020 Nov 11;10:19513. doi: 10.1038/s41598-020-76397-4 (PMC7658243; doi:10.1038/s41598-020-76397-4)
Supplement: Supplementary file 1 — Supplementary material 1 [file 41598_2020_76397_MOESM1_ESM.docx]

**Melanin concentration and depolarization metrics measurement by polarization-sensitive optical coherence tomography: Supplementary information**

**Masahiro Yamanari**^1,*^**, Mutsuki Mase**^1^**, Ryo Obata**^2^**, Mitsuhiro Matsuzaki**^3,4^**, Takahiro**

**Minami**^2^**, Seiji Takagi**^3,4^**, Motoshi Yamamoto**^2^**, Noriko Miyamoto**^3,4^**, Koji Ueda**^2^**, Naoshi**

**Koide**^5^**, Tadao Maeda**^3,4,5^**, Kota Totani**^1^**, Nobuyori Aoki**^1^**, Yasuhiko Hirami**^3,4,5^**, Satoshi Sugiyama**^1^**, Michiko Mandai**^3,5^**, Makoto Aihara**^2^**, Masayo Takahashi**^3,5,6^**, Satoshi Kato**^2^**, and Yasuo Kurimoto**^3,4,5^

^1^Engineering Department, Tomey Corporation, Nagoya, Aichi, Japan

^2^Department of Ophthalmology, Graduate School of Medicine and Faculty of Medicine, The University of Tokyo, Tokyo, Japan

^3^Department of Ophthalmology, Kobe City Eye Hospital, Kobe, Hyogo, Japan

^4^Department of Ophthalmology, Kobe City Medical Centre General Hospital, Kobe, Hyogo, Japan

^5^Laboratory for Retinal Regeneration, Riken Centre for Biosystems Dynamics Research, Kobe, Hyogo, Japan ^6^Vision Care Inc., Kobe, Hyogo, Japan

^*^m-yamanari@tomey.co.jp


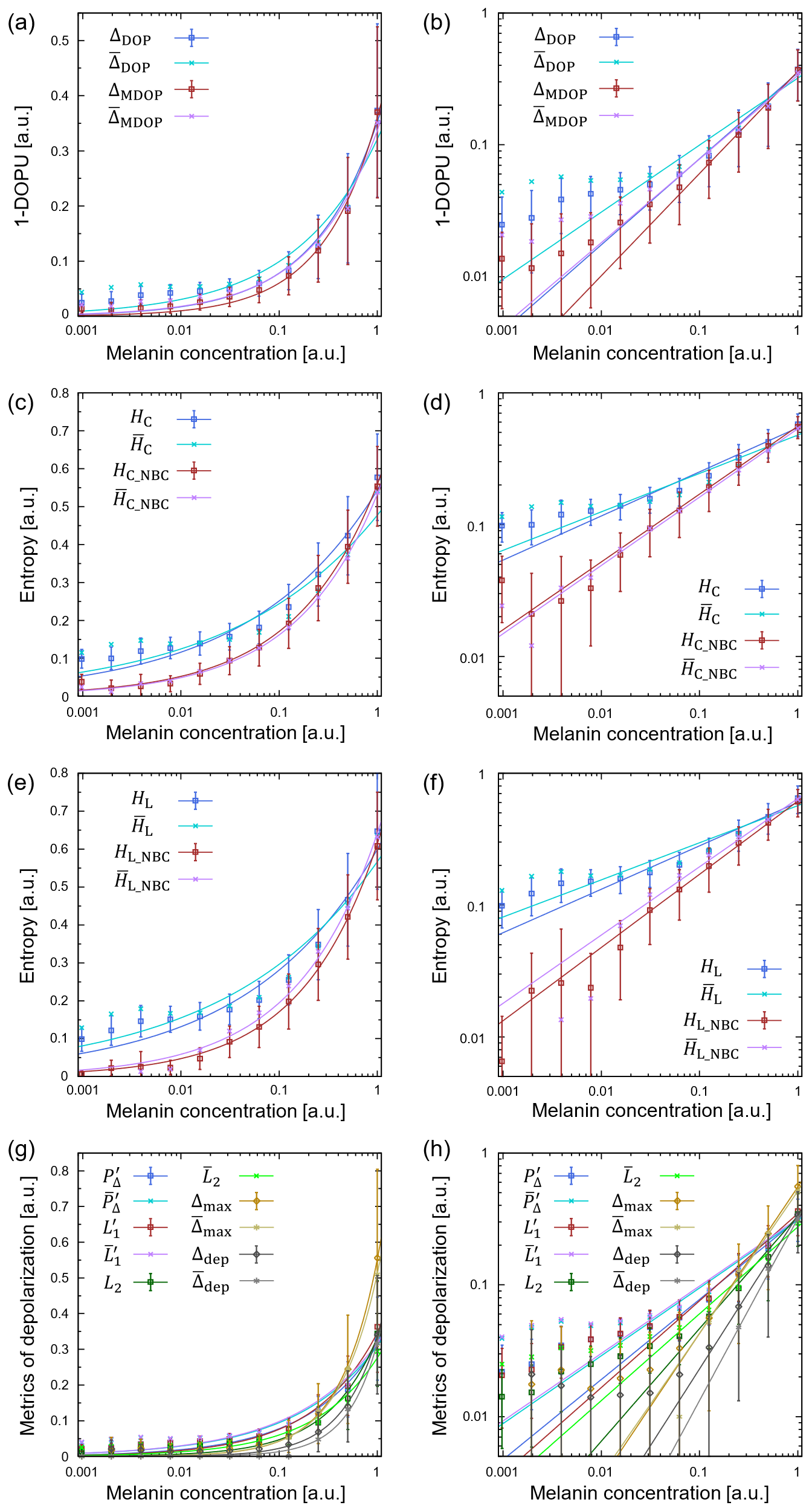


**Supplementary Figure S1.** Plots of melanin concentration versus various depolarization metrics. The plots were same as Fig. 2 except for the curve fitting. Whereas the polynomial fitting was used except for the noise-bias corrected entropy in Fig. 2, we used the exponential fitting using $y={10}^{b}x^{a}$, where *x* and *y* denoted the melanin concentration and the depolarization metrics, respectively, for all data in this figure.

**Supplementary Table S1.** Fitted parameters using $y={10}^{b}x^{a}$, where *x* and *y* denoted the melanin concentration and the depolarization metrics, respectively. RSS stands for the residual sum of squares. The asterisks indicate RSS < 0.001, which has no relation to statistical significance.

|  | *a* | *b* | RSS |
| --- | --- | --- | --- |
| $\Delta_{\mathrm{DOP}}$ | 0.65 | -0.45 | 0.00476 |
| $\bar{\Delta}_{\mathrm{DOP}}$ | 0.51 | -0.49 | 0.00793 |
| $\Delta_{\mathrm{MDOP}}$ | 0.78 | -0.44 | 0.00119 |
| $\bar{\Delta}_{\mathrm{MDOP}}$ | 0.63 | -0.47 | 0.00184 |
| $H_{C}$ | 0.33 | -0.27 | 0.00887 |
| $\bar{H}_{C}$ | 0.29 | -0.32 | 0.02065 |
| $H_{C\_NBC}$ | 0.52 | -0.25 | 0.00094^*^ |
| $\bar{H}_{C\_NBC}$ | 0.52 | -0.27 | 0.00034^*^ |
| $H_{L}$ | 0.33 | -0.22 | 0.01415 |
| $\bar{H}_{L}$ | 0.28 | -0.25 | 0.02355 |
| $H_{L\_NBC}$ | 0.56 | -0.21 | 0.00076^*^ |
| $\bar{H}_{L\_NBC}$ | 0.52 | -0.19 | 0.00727 |
| $P_{\Delta}^{'}$ | 0.61 | -0.50 | 0.00297 |
| $\bar{P}_{\Delta}^{'}$ | 0.51 | -0.51 | 0.00641 |
| $L_{1}^{'}$ | 0.66 | -0.46 | 0.00343 |
| $\bar{L}_{1}^{'}$ | 0.51 | -0.50 | 0.00674 |
| $L_{2}$ | 0.85 | -0.48 | 0.00250 |
| $\bar{L}_{2}$ | 0.66 | -0.56 | 0.00380 |
| $\Delta_{\max}$ | 1.11 | -0.26 | 0.00147 |
| $\bar{\Delta}_{\max}$ | 1.08 | -0.29 | 0.00058^*^ |
| $\Delta_{\mathrm{dep}}$ | 1.17 | -0.47 | 0.00129 |
| $\bar{\Delta}_{\mathrm{dep}}$ | 1.38 | -0.50 | 0.00048^*^ |
